# Computer Simulation of Catheter Cryoablation for Pulmonary Vein Isolation

**DOI:** 10.3390/healthcare12151508

**Published:** 2024-07-30

**Authors:** Solange I. Rivera, Clara P. Bernal, Rafael Martínez-Peláez, Rogelio Robledo-Nolasco, Gerardo De León-Larios, Vanessa G. Félix, Rodolfo Ostos, Gladys E. Maestre, Jesús D. Melgarejo, Luis J. Mena

**Affiliations:** 1 Department of Chemical, Electronic and Biomedical Engineering, Science and Engineering Division, Universidad de Guanajuato, Guanajuato 36250, Mexico; si.rivera@ugto.mx; 2 Faculty of Engineering and Technologies, Universidad La Salle Bajío, Leon 36700, Mexico; clark.bh@hotmail.com; 3 Department of Systems and Computer Engineering, Faculty of Engineering and Geological Sciences, Universidad Católica del Norte, Antofagasta 1270709, Chile; rafael.martinez@ucn.cl; 4 Computer Academic Unit, School of Information Technologies, Universidad Politécnica de Sinaloa, Mazatlan 82199, Mexico; vfelix@upsin.edu.mx (V.G.F.); rostos@upsin.edu.mx (R.O.); 5 Department of Interventional Cardiology and Electrophysiology, Centro Médico Nacional 20 de Noviembre ISSSTE, Mexico City 03100, Mexico; rogelio_robledo@hotmail.com (R.R.-N.); gerardo.deleon86@gmail.com (G.D.L.-L.); 6 Institute of Neuroscience, School of Medicine, University of Texas Rio Grande Valley, Edinburg, TX 78550, USA; gladys.maestre@utrgv.edu (G.E.M.); jesus.melgarejo@utrgv.edu (J.D.M.)

**Keywords:** cryoablation, catheter therapy, atrial fibrillation, pulmonary veins, computer simulation

## Abstract

Cryoablation is a well-established medical procedure for surgically treating atrial fibrillation. Cryothermal catheter therapy induces cellular necrosis by freezing the insides of pulmonary veins, with the goal of disrupting abnormal electrical heart signals. Nevertheless, tissue damage induced by cold temperatures may also lead to other complications after cardiac surgery. In this sense, the simulation of catheter ablation can provide safer environments for training and the performance of cryotherapy interventions. Therefore, in this paper, we propose a novel approach to help better understand how temperature rates can affect this procedure by using computer tools to develop a simulation framework to predict lesion size and determine optimal temperature conditions for reducing the risk of major complications. The results showed that a temperature profile of around −40 °C caused less penetration, reduced necrotic damage, and smaller lesion size in the tissue. Instead, cryotherapy close to −60 °C achieved a greater depth of temperature flow inside the tissue and a larger cross-section area of the lesion. With further development and validation, the framework could represent a cost-effective strategy for providing personalized modeling, better planning of cryocatheter-based treatment, and preventing surgical complications.

## 1. Introduction

In recent decades, emerging computer technologies have played a critical role in modern medicine and life sciences [1]. Computer solutions based on the integration of machine learning, mobile computing, and modeling have provided a more complete approach to healthcare. This has improved the accuracy and effectiveness of prevention, diagnosis, and treatment strategies for chronic disorders [2,3,4].

In cardiovascular science, computational models and monitoring mobile systems have been developed to design patient-specific modeling for cardiac surgery [5] and to record cardiovascular health data at anytime and anywhere [6]. Moreover, computed tomography scans and magnetic resonance imaging have provided a more detailed assessment of the heart [7], and image-guided interventions provide real-time information to conduct more precise and safer cardiac procedures [8].

On the other hand, computer simulations have also been used to improve clinical practice in low-resource settings [9]. The development of simulated environments has contributed to improving the quality of cardiology training programs [10,11], increasing the effectiveness of therapy [12], reducing risks for patients [13], and enhancing the performance of surgical interventions [14]. For the assessment and surgical treatment of heart-valve disease, patient-specific computer simulation may improve the planning and results of catheter-based treatment [15,16].

Atrial fibrillation (AF) is the most common clinically relevant cardiac arrhythmia, affecting an estimated 33.5 million people worldwide [17]. The disease is more prevalent among older adults [18] and it is one of the leading causes of stroke, sudden death, and cardiovascular morbidity and mortality in the world [19]. AF is a supraventricular tachycardia characterized by disorganized atrial activation and consequent impairment of atrial mechanical function, with focal origins in the pulmonary veins (PVs) [20]. Over the past 20 years, the rates of individuals with AF and hospitalizations attributed to AF have been increasing; this is probably due to the fast aging of the population [21]. In Mexico, studies have reported an increased risk of cerebral and systemic embolism attributed to AF [22], as well as in the incidence of the disease in the population [23].

The treatment for AF aims at controlling sinus rhythm and heartrate rhythms [24], which are initially achieved with the use of an oral anticoagulant and antiarrhythmic drugs [25]. However, in addition to monitoring the side effects of these medications [24,26], complete elimination of persistent AF is frequently unsuccessful with only oral pharmacological agents [24]. Therefore, catheter ablation therapy has emerged as the most effective alternative for surgically treating AF [26,27]. This medical procedure uses thermal energies to create small scars inside the PVs, disrupting the electrical signals that cause AF. Radiofrequency (RF) and cryothermal are the most widely used energy sources for performing cardiac ablation [27,28,29,30]. Despite RF being considered a gold standard method [26,27], RF ablation converts electromagnetic energy to thermal energy [31], which results in the heating and destruction of tissue [32]. In addition, the size of tissue damaged correlates with the amount and duration of the energy delivered to produce heat on the tissue [31,32]. Thus, high power and larger time periods of RF ablation have been associated with cardiac perforations [33], pericardial effusion/tamponade [34], and PV stenosis [35].

PV isolation achieved by cryoablation (CBA) has similar effectiveness to RF for treating AF as it is a shorter procedure and has lower rates of major complications [26,27,30,36]. In CBA, cryothermal energy applied with a balloon leads to cellular necrosis by freezing the target region [37,38,39]. CBA is now considered a well-established and safe treatment option to perform circumferential isolation of the PVs [27,40]. The most common complication associated with CBA is phrenic nerve palsy [27,41,42], which can be avoided by reducing the total freezing time [43], implementing compound motor action potential monitoring [44], or immediate balloon deflation [45]. To avoid such complications, computerized simulations can provide safer CBA environments for reducing surgical complications and preventing irreversible damage to the atrium or PVs [45,46]. Some computer models have been developed to improve the knowledge of the biophysics behind AF ablation [47]. These computerized models have evaluated how heterogeneous ventricular tissues influence lesion size [48], how blood motion affects the cardiac chamber [49], how tissue surface can be modeled based on data generated by the RF ablation [50], and how to represent different electrode–tissue contact force values [51]. 

For CBA therapies, a computer cryoballoon model has been proposed that uses electromagnetic and thermal simulations to estimate the temperature profile that may be used to optimize AF ablation [52,53]. Nevertheless, no specific criteria have been established to select an appropriate temperature rate during the CBA procedure. Some computational models have predicted a minimum duration of freezing to obtain a durable and transmural lesion of cardiac tissue [54]. In addition, other parameters, such as cryoballoon position, seem to also affect the predicted effectiveness of the procedures [55]. To the best of our knowledge, there is not a computer-model study that addresses the estimation of an adequate temperature profile for CBA, based on lesion simulation in cardiac tissue. Therefore, in this paper, we proposed a novel simulation framework approach to (1) help better understand how temperature rates can affect CBA procedure; (2) represent adequate lesion size; (3) guide better CBA dosing strategies; and (4) determine optimal temperature conditions for reducing the risk of major complications. 

## 2. Materials and Methods

### 2.1. Software

For modeling and simulation of the CBA procedure, we used COMSOL Multiphysics software (v5.4), which is a general-purpose simulation software that provides a user-friendly environment to analyze a wide range of physical phenomena [54] including CBA treatments [53]. The Heat Transfer module [54] was used to model operational conditions during freezing and thawing phases. This tool includes a library for bioheating applications [54] that allows accounting for the influence of various processes in living tissue as contributions of heat exchange between a structure and its surroundings (heat flow) and flow direction of heat per unit of area (heat flux). Laminar flow interface [54] was also used to compute specific physical parameters of blood fluid in PVs, such as dynamic viscosity, flow pressure, and velocity.

### 2.2. Simulation Parameters

Computer simulations were performed for four materials according to the thermal and physiological properties described in Table 1. Mean body temperature of 36.7 °C [56], and volumetric blood perfusion of 0.012 mL s^−1^ cm^−3^ [57], were predefined. In addition, a heat flux of −151,924.04 joules was applied toward the polyurethane balloon equivalent to minimal internal temperature fixed at −80 °C [58]. Metabolic heat generation for solid material (polyurethane) was not considered in the simulation because specified solids are homogeneous and cause blood perfusion but no significant metabolic activity [59].

Temperature response of biological tissue due to the heat conduction in PVs by blood perfusion was modeled using the Pennes bioheat transfer equation [60], because lesion size is affected by the intramyocardial blood flow [61]. Thus, this equation is widely used to solve the temperature distribution between tissue and blood for thermal therapies [62]:

(1) ρc ∂T/∂t=∇ (k ∇T)+Wb Cb (Ta−T)+Qm

where *ρ* is tissue density, *c* is specific heat, *k* is thermal conductivity, *Cb* is the specific heat of blood, *Ta* is arterial blood temperature, *T* is ambient temperature, *∂T/∂t* is the rate of temperature variation, *Wb* is the blood flow velocity per unit volume, and *Qm* is metabolic heat generation. Equation (1) was applied for each of the materials used in the implementation of the three-dimensional model, with the parameters shown in Table 1.

### 2.3. Dataset

Data to estimate thermal conductivity (*k*) for different PVs were collected from therapy logs of five patients (mean age 55.7 years; 60% women) of the hemodynamics unit of Centro Medico Nacional 20 de Noviembre in Mexico City. Informed consent was obtained for all subjects. The ethical review board of Centro Medico Nacional 20 de Noviembre approved the protocol.

Table 2 shows time (seconds) and temperature records of patients undergoing CBA therapy for isolation of the PVs. *TZ* is the time when the thermocouple indicated a temperature of zero degrees Celsius (°C), *TI* is the time when it reached −30 °C, *TM* is the time when the cryoballoon reached the maximum cold temperature (time to isolation) in each specific CBA procedure, and *TT* is the total time of cryotherapy, including when the tissue returned to its initial temperature after the thawing phase.

### 2.4. Simulation Model

**Figure 1:**
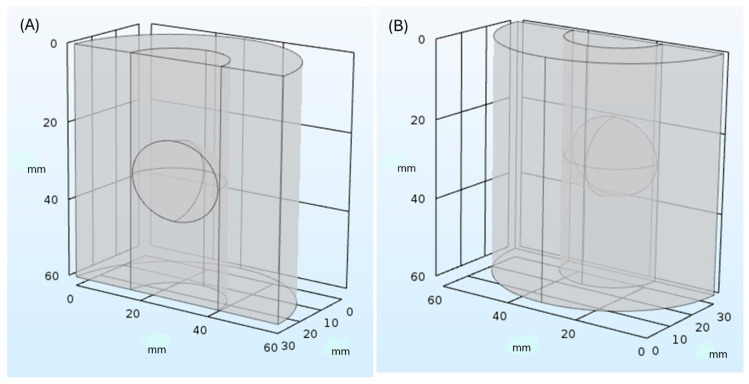
Geometric model. (**A**) Frontal view and (**B**) back view.

For designing and developing the simulation models, we specifically used the COMSOL Multiphysics module of Heat Transfer in Solids (ht) [54], with the aim of modeling tissue cooling based on convection heat exchange between solid synthetic material (polyurethane of cryoballoon) and solid biological tissue (vascular wall of PV). The geometry of the computational model consisted of three concentric cylinders with a height of 60 mm. The innermost cylinder replicated the vascular lumen of PVs, a hollow passageway through which blood flows [63]; the second cylinder simulated pulmonary tissue; and the outermost cylinder represented the vascular wall pulmonary tissue in contact with the exterior. The first cylinder (Figure 1) included an ellipsoid with diameters of 23 mm and height of 20 mm to approximately represent second-generation cryoballoon Medtronic Arctic Front Advance, with an inflated balloon diameter of 23 mm [64].

Using previous normal values established for diameter and thickness of PVs [65,66], we calculated the average value to define simulation parameters for each PV (Table 3).

Simulation was performed by each patient with a balloon inflated to 23 mm. Two freezing/thawing cycles were administered in each case to ensure representation of effective therapy. Depth of temperature flow was used to measure changes and penetration of cold through tissue, for estimating lesion formation size as temperature increases during CBA treatment.

### 2.5. Estimation of Lesion

**Figure 2:**
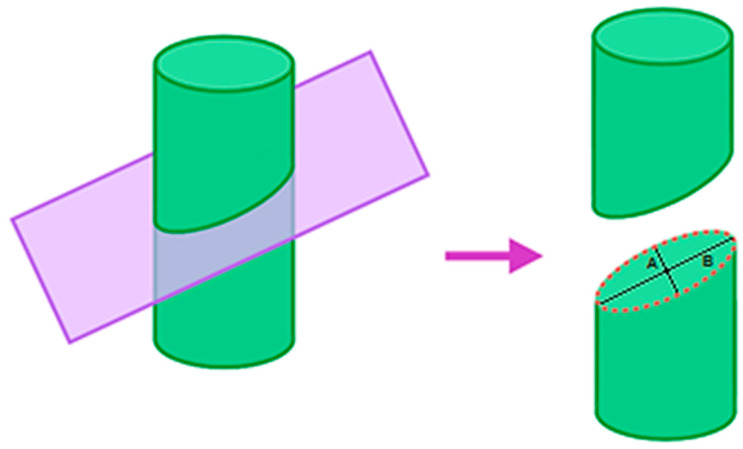
Lesion cross-section area.

For representing thermal lesion cross-section area during the CBA procedure, we proposed Equation (2); using the ellipse area formula by considering lesion morphology as an elliptical shape [51] (see Figure 2); estimating lesion depth (*A*) by multiplying by 2 the highest distance between cryoballoon and the outermost border of pulmonary tissue; and defining lesion width (*B*) as a constant of 23 mm according to the diameter of the inflated balloon. This takes into account the zone of cooling around the equator of the second-generation cryoballoon extended, producing more uniform lesions independent of the position of the balloon [67].

(2) $$Lesion\;Area=\pi \times 2\left(\frac{A}{2}\right)\times \left(\frac{B}{2}\right)\;\;\;\;\;\;\;\;$$

where A is lesion depth and B is lesion width.

### 2.6. Temperature Parameters

Conversion of Kelvin to Celsius temperature was calculated by subtracting 273.15 from the Kelvin temperature. Temperature cooler than −15 °C was considered the limit to start measuring the depth and width of the lesion, because ice crystals form exclusively in the extracellular space as the tissue temperature drops below this threshold [38].

## 3. Results

### 3.1. Temperature Distribution

Three-dimensional computational model tracked changes in temperature over time during CBA is illustrated in Figure 2. The temperature distribution is represented using a color scale. Each different color line indicates temperature changes in the target region. The green color corresponds to a temperature of 0 °C, which will be used to determine the lesion tissue boundary. Our results demonstrate that temperature was evenly distributed in the model during the first 10 s of each therapy. The direction of the heat flow was reflected negatively since the cryocatheter acted as a heat absorber inside the tissue. Heat flow moved from the hotter to colder areas as it approached the cryoballoon. A heat flow of −7.84 joules was observed during the simulation for predefined values, with an internal cryoballoon temperature of −80 °C, tissue temperature of 36.7 °C, and heat flux of −151,924.04 joules.

### 3.2. CBA Simulation

**Figure 3:**
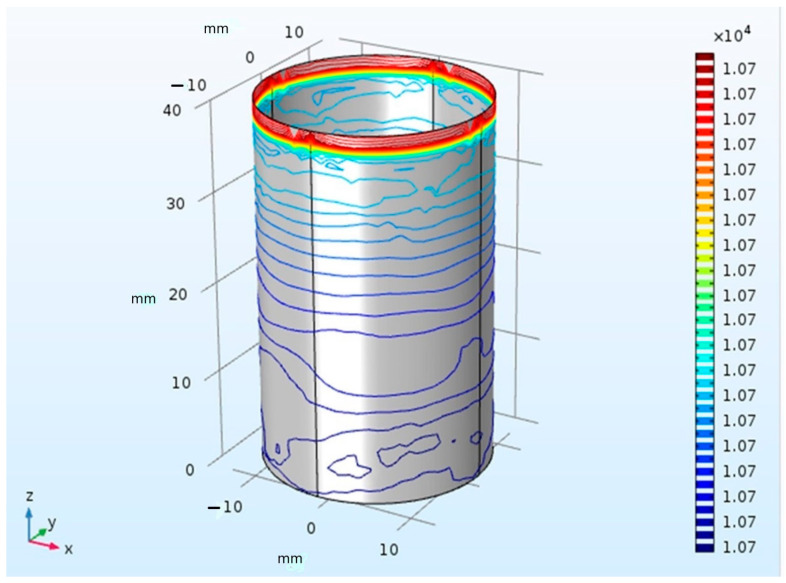
Computational model for temperature distribution during cryotherapy.

During the simulation of tissue freezing for five patients undergoing cryotherapy (Figure 3), the temperature distribution on the front face of the geometric model was nonuniform after 240 s. Most therapies reached 0 °C after the initial 10 s of localized cooling, resulting in tissue damage on the contact area with the cryoballoon and at the periphery due to hypothermia. Maximum freezing temperatures for PV isolation were reached in an average time of 35.20 ± 3.54 s.

The model also monitored the moment when the thermocouple registered −30 °C, because this temperature usually results in vasoconstriction as an initial response of the cells to the application of ice [68]. It is important to note that at temperatures between 0 °C and −15 °C, the majority of cells exhibit unique resistivity to freezing for short periods because the cell membrane acts as a barrier to ice crystal nucleation [69]. Therefore, ice crystal formation began in the extracellular space at −15 °C (heterogeneous nucleation) [38], creating initial tissue damage. As the temperature dropped between −20 °C and −30 °C, vascular equilibrium was reached and ice crystal formation in the intracellular space was observed, resulting in increased tissue injury. This situation occurred because the ice crystals compressed and deformed the nucleus, and cytoplasmic components led to permanent cellular dysfunction. On the other hand, for cryogenic therapy close to −40 °C, the formation of clusters of ice crystals was observed inside and outside the cell (homogeneous nucleation) [69]. This phenomenon resulted in irreversible disruption of organelles and the cell membrane, and led to cell death through apoptosis.

**Figure 4:**
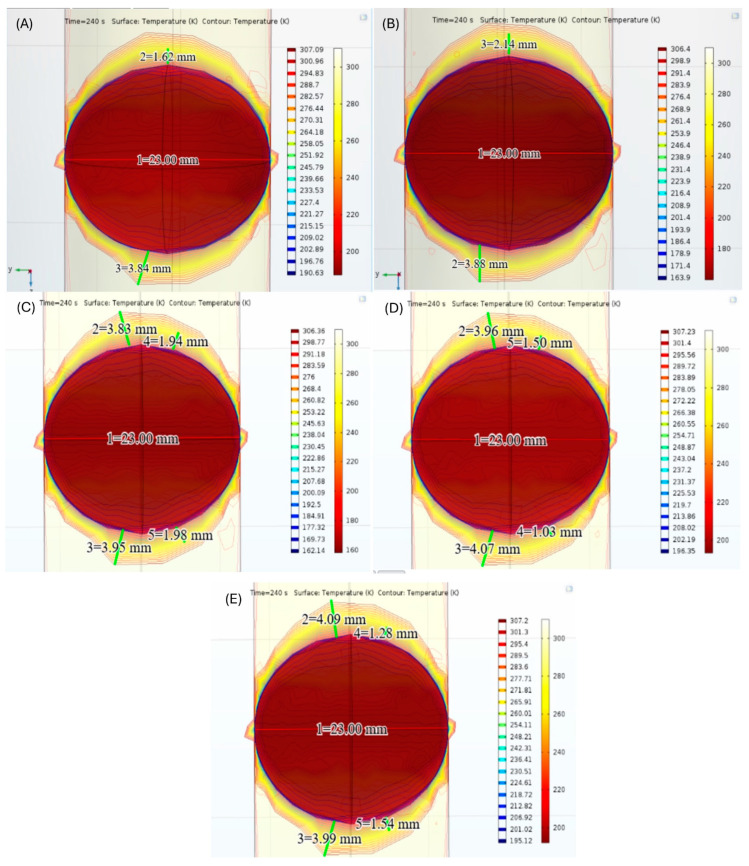
Results of CBA simulation for five patients (**A**–**E**) undergoing cryotherapy, including the highest distances from the cryoballoon to the vascular wall and to the outermost border of pulmonary tissue.

Finally, we show, in Figure 4 and Table 4, relevant measurements of each patient corresponding to lesion size and temperature flow inside tissue after cryotherapy. Higher distances between the cryoballoon and the vascular wall indicated a lower value of temperature flow depth and, therefore, less penetration of cryothermal energy into the tissue. The average of lesion depth predictions for simulated ablation was 3.51 ± 0.51 mm, which is in agreement with previous in vivo performance studies of the second-generation cryoballoon (3.02 ± 1.13 mm) [70].

## 4. Discussion

This study generated simulations of thermal propagation by freeze application inside tissue during cryotherapy. This method allowed us to assess the effectiveness of CBA procedures based on lesion formation size, temperature changes through tissue depths, and reduction in surgical complications. The simulation framework provided a basic approach to geometrically model lesions inside PVs by tracking thermodynamic changes over time and estimating tissue injury as a response to freezing on contact with tissue at 36 °C. Computer simulation incorporated the Pennes bioheat equation to model the effect of heat transfer by blood perfusion in PVs while the tissue is in a nonfrozen state. Additionally, relevant time and temperature conditions of real clinical practice of CBA were also added to the model.

After analyzing the model predictions in relation to tissue damage with respect to vascular wall temperature after 240 s of therapy, our simulations indicated that at approximately −40 °C there was lower penetration of temperature into tissue. This resulted in less lesion depth, reduced necrotic tissue, and smaller lesion size. On the other hand, in the case of temperatures close to −60 °C and higher profundity of temperature flow inside the tissue, the simulations indicated a larger depth of the lesion. Overall, CBA procedures were considered successful because the formation of cluster-like ice crystals was also evident for this temperature range.

Our simulations also indicated that a temperature profile around −40 °C could enhance cellular self-support against crystal formation, allowing greater effectiveness in cryogenic therapy because ice crystal formation by extreme freezing (temperature colder than −50 °C) can cause abrasive action on the tissue, mechanically disrupting cardiac cells, and ultimately leading irreversible tissue damage [38,71]. On the other hand, this temperature profile could help to reduce phrenic nerve injury occurring at lower temperature rates (mean −49.5 °C; median −50 °C) [72] as the phrenic nerve temperature is strongly associated with the durability of phrenic nerve damage [73].

Based on our work, we propose a novel computer framework to predict CBA lesions from real patient data and better guide future studies for dosing cryoenergy transfer by using progressive cooling from −30 °C (intracellular ice crystal formation) to below –50 °C (permanent tissue damage). Favorable outcomes in CBA procedures based on computational simulations may improve the safe planning of CBA treatments and optimize costs by performing in vivo clinical trials [74]. To the best of our knowledge, this is the first CBA computational model that adequately represents lesion size, estimates an appropriate temperature profile to reduce clinical complications, proposes a temperature range for a better dosing strategy of freezing, and uses multiple data from patients with informed consent.

### Limitations

Our study should be interpreted within the context of its limitations. First, this study included a small data sample due to the difficulty of obtaining informed consent, derived from limited health literacy [75] and a low level of comprehension of our study population about the informed consent process [76]. Nevertheless, previous CBA simulation studies do not report evidence of real data [52,53]. On the other hand, analytical predictive studies may provide more truthful results with a small sample, because modeling parameters can be controlled with higher precision [77]. Second, regarding the construction of our models and simulations, geometric modeling included perfectly concentric cylinders and ellipsoids to represent PVs and the cryoballoon. Moreover, simulation parameters for heat transfer during vascular perfusion were defined by assuming homogeneous tissues and suitable balloon positioning. Therefore, although these assumptions contributed to addressing model complexity, these could decrease the precision of simulating interactions among ice crystal formation, blood flow, and heat transfer on cardiac tissue, and subsequently predict lesion formation during the CBA procedure. Furthermore, modeling was performed with a second-generation cryoballoon (Arctic Front Advance). A fourth-generation design (Arctic Front Advance Pro) is currently available with significant improvements in cold transfer that capture temperatures and time-to-isolation monitoring [53,78]. This is a dilemma every model faces to achieve an appropriate balance between accuracy and simplicity [79].

## 5. Conclusions

We developed a computer simulation framework to estimate the optimal temperature profile according to CBA lesion formations. The model confirmed that the efficacy of the procedure is sensitive to freeze rates. Our findings indicate that temperatures around −40 °C could reduce collateral damage and irreversible cardiac tissue injury. With further development and validation, the framework could represent a cost-effective strategy for providing personalized modeling for CBA, better planning of cryocatheter-based treatment, and prevention of major surgical complications.

## Figures and Tables

**Table 1:** Thermal and physiological properties of the materials used for simulation.

Material	Thermal Conductivity (W/mK)	Specific Heat (J/kgK)	Tissue Density (kg/m^3^)	Temperature
Blood	0.54	4000	1057	310.15
Vascular wall	1.3	3600	1200	310
Polyurethane	0.02	1500	1110	198.15
Pulmonary tissue	0.03	2963	1000	311

**Table 2:** Time in seconds and temperature records for patients undergoing CBA therapy.

Patient 1	Patient 2	Patient 3	Patient 4	Patient 5
*TZ* (0 °C) = 10	*TZ* (0 °C) = 8	*TZ* (0 °C) = 12	*TZ* (0 °C) = 12	*TZ* (0 °C) = 13
*TI* (−30 °C) = 23	*TI* (−30 °C) = 29	*TI* (−30 °C) = 36	*TI* (−30 °C) = 30	*TI* (−30 °C) = 31
*TM* (−60 °C) = 32	*TM* (−42 °C) = 33	*TM* (−41 °C) = 42	*TM* (−63 °C) = 34	*TM* (−43 °C) = 35
*TT* = 240	*TT* = 240	*TT* = 240	*TT* = 240	*TT* = 240

**Table 3:** Simulation parameters for pulmonary veins.

Pulmonary Vein	Ostium Diameter (mm)	Vascular Wall Thickness (mm)
Right superior	18	2.7
Right inferior	12	1.8
Left superior	19	2.8
Left inferior	13	1.9

**Table 4:** Results of computer simulation for lesion formation and penetration of temperature through tissue depths after CBA.

Maximum Freezing Temperatures (°C)	Distance between Cryoballoon and Vascular Wall (mm)	Lesion Depth (mm)	Lesion Area (mm^2^)
−60	3.88	4.28	77.28
−42	3.84	3.24	58.50
−41	4.07	3.00	54.17
−61	3.95	3.96	71.50
−43	4.09	3.08	55.61

## Data Availability

The original contributions presented in the study are included in the article, further inquiries can be directed to the corresponding author.
